# Genome-Wide Analysis of MAMSTR Transcription Factor-Binding Sites via ChIP-Seq in Porcine Skeletal Muscle Fibroblasts

**DOI:** 10.3390/ani13111731

**Published:** 2023-05-23

**Authors:** Chenlei Li, Zhe Zhang, Yilin Wei, Kunlong Qi, Yaqing Dou, Chenglei Song, Yingke Liu, Xinjian Li, Xiuling Li, Kejun Wang, Ruimin Qiao, Feng Yang, Xuelei Han

**Affiliations:** College of Animal Science and Technology, Henan Agricultural University, Zhengzhou 450002, China

**Keywords:** meat quality traits, skeletal muscle development, chip-seq, *MAMSTR* gene

## Abstract

**Simple Summary:**

Skeletal muscle is the most abundant tissue in animals, accounting for 45–60% of the body weight of meat animals, and the growth and development of skeletal muscle has the most direct impact on lean meat percentage and muscle quality. Therefore, exploring the molecular regulation mechanism of skeletal muscle growth and development is an important basis for improving pig meat production performance, and it has also been a research focus in animal genetics in recent years. In this study, ChIP-seq and other methods were used to explore the transcriptional regulation mechanism of new transcription factor *MAMSTR* in pig skeletal muscle development, The results of this study can further reveal the regulatory mechanism of pig skeletal muscle growth and development, identify new genes related to pig skeletal muscle growth and development, and provide a better reference for pig-farming practices and breeding of high-quality meat pig breeds.

**Abstract:**

Myocyte enhancer factor-2-activating motif and SAP domain-containing transcriptional regulator (*MAMSTR*) regulates its downstream through binding in its promoter regions. However, its molecular mechanism, particularly the DNA-binding sites, and coregulatory genes are quite unexplored. Therefore, to identify the genome-wide binding sites of the *MAMSTR* transcription factors and their coregulatory genes, chromatin immunoprecipitation sequencing was carried out. The results showed that *MAMSTR* was associated with 1506 peaks, which were annotated as 962 different genes. Most of these genes were involved in transcriptional regulation, metabolic pathways, and cell development and differentiation, such as AMPK signaling pathway, TGF-beta signaling pathway, transcription coactivator activity, transcription coactivator binding, adipocytokine signaling pathway, fat digestion and absorption, skeletal muscle fiber development, and skeletal muscle cell differentiation. Lastly, the expression levels and transcriptional activities of *PID1*, *VTI1B*, *PRKAG1*, *ACSS2*, and *SLC28A3* were screened and verified via functional markers and analysis. Overall, this study has increased our understanding of the regulatory mechanism of *MAMSTR* during skeletal muscle fibroblast development and provided a reference for analyzing muscle development mechanisms.

## 1. Introduction

Skeletal muscle is the most abundant tissue in the animal body, accounting for 45–60% of the weight of meat animals. It has the most direct effect on lean meat rate and muscle quality; therefore, exploring the molecular regulation mechanism of skeletal muscle growth and development is necessary, and it has been a research hotspot and focus of animal genetics in recent years [1,2,3,4]. The development of skeletal muscle cells is a multistage and highly ordered biological process (BP), which is regulated by the programmed expression of a series of important genes and then regulates the expression of skeletal muscle-specific genes [5,6]. The study of the molecular process of pig myogenesis not only contributes to the understanding of the myogenesis process and provides animal models for the study of human diseases, but also offers new materials for the genetic improvement of pig growth traits and muscle quality. In recent years, with the emergence of high-throughput technologies, such as gene chip and SAGE technology, the study of the regulation of pig muscle gene expression has been accelerated [7,8,9]. During skeletal muscle development, the programmed expression of a series of important genes leads to the expression of skeletal muscle-specific genes, which form complex regulatory networks and signal transduction pathways to promote skeletal muscle development in an orderly and controllable way [10]. The regulation of gene expression is an important content of functional genomic research, and the regulation of transcription level is the most important first step in the process of gene expression and one of the main ways for gene expression regulation.

Myocyte enhancer factor-2 (MEF2)-activating motif and SAP domain-containing transcriptional regulator (*MAMSTR*) belongs to the myocardin transcription factor (TF) family [11] because of its SAP domain-containing structure, including a SAP structure that belongs to the domain TF family. The SAP structure domain is a conservative motif formed by 33 amino acid residues, which is present in many nuclear proteins and can activate the expression of myo-specific reporter genes [12]. Another characteristic is that the amino terminal contains an MEF2-binding sequence, which can activate MEF2. MEF2 belongs to the family of transcriptional regulation factor by MADS-box, is widely expressed in muscle and nerve tissues, and plays an important role in the development of the nervous system and muscles, such as mediated skeletal muscle, in the differentiation of cardiac and smooth muscle cells, and in gene transcription for control muscle cell differentiation [13,14]. Creemers et al. found that *MAMSTR* is located in chromosome 7 in mice, encoding 421 amino acids, in the skeletal muscle, brain, placenta, and spleen of mice and humans, and within the nucleus *MAMSTR*; expressed *MEF2C* parts overlap, which thus increases the *MEF2C* transcriptional activity [11]. Meadows identified that the *MAMSTR* gene is highly expressed in skeletal muscle during embryonic development and adulthood stage and can increase the expression of skeletal muscle genes by activating myogenic regulatory factors (MRFs) in the African claw toad. Previous studies have also shown that the *MAMSTR* gene plays an important role in the regeneration and reconstruction of skeletal muscle by enhancing the expression of *MYOD1* in muscle-specific enhancement factor by interacting with *MEF2* and myocardin genes. Mokalled found that *MAMSTR* enhances the expression of *MYOD1*, a muscle-specific enhancement factor, by interacting with *MEF2* and myelin families and plays an important role in the regeneration and reconstruction of skeletal muscle [15].

Several candidate genes related to meat quality and growth rate in pigs have been identified and used for breeding, such as *RN* [16], *RYR1* [17], *H-FABP* [18], and *IGF2* [19]. However, substantial potential candidate genes still need to be discovered and studied. Chromatin immunoprecipitation, followed by next-generation sequencing, is an effective technique for the identification of specific TF-binding sites (TFBSs). Moreover, chromatin immunoprecipitation sequencing (ChIP-seq) offers less noise and larger coverage with high resolution than its array-based predecessor ChIP-chip [20]. ChIP-Seq has become an essential technique for examining gene regulation, TFBSs, histone modification, and DNA methylation [21,22]. It has the advantages of high cost effectiveness, efficiency, detection sensitivity, and coverage, and is a key technology in the field of gene expression regulation. At present, limited knowledge exists about the porcine *MAMSTR* gene. Therefore, we investigated the role of *MAMSTR* in skeletal muscle fibroblasts via ChIP-Seq analysis in order to study the involvement of downstream genes associated with skeletal muscle development. This study will help expand our understanding of the regulatory role of *MAMSTR* during skeletal muscle development in pig.

## 2. Materials and Methods

### 2.1. Porcine Myofibroblast Culture

Culture of porcine myofibroblasts: The longissimus dorsi muscles were isolated from four purebred Large White piglets (1 d), with an ophthalmic scissors of tissue piece about the size of 1 mm^3^, and a suitable amount of 0.25% pancreatic enzymes (containing 0.04% EDTA) was added. Then, the temperature was kept at 37 °C for digestion in 20 min, and a cell sieve filter was used for collecting filtrate. The filtrate was centrifuged for 5 min at 1000 r/min and discarded, and the fibroblast group was obtained. It was washed with PBS, centrifuged for 5 min at 1000 r/min, and discarded, and a relatively pure cellule was obtained. Afterward, a suitable amount of a complete medium (DMEM + 10% FBS) was added, and full beat blending in a Petri dish was performed using a 37 °C and 5% CO_2_ incubator for training. C2C12 cell and PK-15 cell line were obtained from the China Center for Type Culture Collection. The PK-15 cell line was cultured in DMEM (Invitrogen, Carlsbad, CA, USA) supplemented with 10% fetal bovine serum (Invitrogen), 100 U/mL of penicillin, and 100 µg/mL of streptomycin (Invitrogen), as recommended by the supplier. C2C12 cells were inoculated in a six-well plate 1 day before transfection and cultured until 50–80% cell growth was fused for transfection.

### 2.2. Recombinant Adenovirus Packaging and Infection

The coding sequence (CDS) region of the porcine *MAMSTR* gene was obtained via reverse transcription-PCR (RT-PCR) and subcloned into the Not I-EcoR I site of the pHBAd-MCMV-GFP vector, and the 3xFlag sequence was added after MAC to construct the adenovirus vector. The human embryonic kidney HEK293 cell line was used to prepare adenovirus. Recombinant adenovirus vector plasmid *MAMSTR* (2 µg) and scaffold plasmid phbad-bhg (4 µg) were transfected with a Lipofiter TM transfection reagent (15 µL). A fresh cell culture was replaced 6 h after transfection. We observed for the signs of virulence of cells every day (the virulence phenomenon refers to the condition in which the cells become larger and round, akin to grapes, and begin to present obvious plaque) and waited for most of the cells to become diseased and shed from the bottom to collect virulence. When the virus came out, the suspension of the diseased cells was harvested and centrifuged at 2000 rpm for 5 min. The supernatant was discarded and added to 6 mL of ST buffer (culture solution + 10% serum + 2.5% glycerin). The virus titer was measured, and the recombinant adenovirus was stored at −80 °C until infection. Skeletal muscle cells were transferred to a six-well plate culture at 1 × 10^5^/well and infected with a MOI = 100 gradient. We set up three groups (Ad-MAMSTR, Ad-GFP, and blank control), and adenovirus infection was performed when cells reached 80% density. The cell medium was changed after 4 h infection, and fluorescence observation was carried out under an inverted fluorescence microscope after culture in an incubator at 37 °C with 5% CO_2_ for 42 h. We then collected the cell and extracted the RNA and protein, and the expression of MAMSTR was detected using qPCR and Western blot (WB).

### 2.3. ChIP Library Preparation and Sequencing

The DNA (10 ng) of each sample was converted to be phosphorylated blunt-ended with T4 DNA polymerase, Klenow polymerase, and T4 polymerase (NEB). An “A” base was added to the 3′ end of the blunt phosphorylated DNA fragments by using the polymerase activity of Klenow (exo minus) polymerase (NEB). Illumina’s genomic adapters were ligated to the A-tailed DNA fragments. PCR amplification was performed to enrich ligated fragments using Phusion High Fidelity PCR Master Mix with HF Buffer (Finnzymes Oy). The enriched product of ~200–700 bp was cut out from gel and purified. The library was denatured with 0.1 M NaOH to generate single-stranded DNA molecules, loaded onto channels of the flow cell at 8 pM concentration, and amplified in situ by using a TruSeq Rapid SR cluster kit (#GD-402-4001, Illumina, San Diego, CA, USA). Sequencing was carried out by running 100 cycles on Illumina HiSeq 2000 in accordance with the manufacturer’s instructions.

### 2.4. Peak Calling and Data Analysis

After the sequencing platform generated the sequencing images, the stages of image analysis and base calling were performed using Off-Line Basecaller Software (OLB V1.8). After passing a Solexa CHASTITY quality filter, the clean reads were aligned to Sus scrofa reference genome using BOWTIE (V2.1.0). Aligned reads were used for peak calling of the ChIP regions via MACS [23] V1.4.0. Statistically significant ChIP-enriched regions (peaks) were identified through Ad-MAMSTR-IP/Ad-MAMSTR-Input using a *p*-value threshold of 10^−5^. Then, the peaks located within −2 Kb to +2 Kb around the corresponding gene transcriptional start site (TSS) were annotated using the UCSC RefSeq database. Motif analysis was performed on the basis of the location of detected peaks and enrichment positions using MEME software [24]. To test whether *MAMSTR* has a differential preference of interaction over the different chromatin regions, we compared their presence in specific chromatin regions, namely promoters (until 3000 bp upstream of TSSs), proximal downstream regions (until 300 bp downstream of the transcriptional termination sites (TTSs)), 5′-untranslated regions (UTRs), 3′-UTRs, exons, introns, and distal intergenic regions. We also used the web-based ChIP-Seq analysis tool ChIPseek [25] to realize genomic annotation and visualization of *MAMSTR* TFBSs.

### 2.5. Gene Ontology (GO) and KEGG Pathway Enrichment Analysis for Peak-Associated Genes

To analyze the primary biological functions, all peak-associated genes were mapped to the GO database (http://www.geneontology.org/, accessed on 20 October 2022.) to categorize the genes or their products into different components, i.e., BP, cellular component, and molecular function, with a fold change ≥ 2 and a false discovery rate (FDR) threshold ≤ 0.001 as a significant enrichment. The KEGG pathway with a corrected *p*-value < 0.05 was defined as a significantly enriched pathway [26]. The Blast2GO program (v2.5.0) was used to obtain GO annotation, and the WEGO software (http://wego.genomics.org.cn/, accessed on 22 October 2022.) was utilized for functional classification. Cytoscape software (v.3.7.2) [27] provided with ClueGO [28] plugin (v.2.5.8) was used to tag the function of these genes and identify the enriched GO terms and KEGG pathway.

### 2.6. RNA Extraction, RT-PCR, and ChIP-qPCR

Total RNA from cells was extracted using a High Pure RNA Isolation kit (Roche, Basel, Switzerland). cDNA was obtained from 1 μg of RNA by SuperScript VILO cDNA synthesis (Invitrogen) in accordance with the manufacturer’s instructions. ChIP-qPCR was performed using the SYBR Green PCR Master Mix and an ABI Prism^®^ 7900HT instrument (Applied Biosystems^®^, Waltham, MA, USA). Primers were designed using OligoPerfect Designer™ (Invitrogen), and reactions were performed in triplicate. The relative amount of each amplified fragment was estimated with respect to the amplification obtained from input DNA and corrected by *GADPH* expression via the 2^−ΔCt^ method. The primer sequences used for qPCR assessment of the mRNA levels of target genes are listed in Table S1.

### 2.7. Gene Promoter Cloning and Luciferase Assays

The core promoter sequence of potential candidate genes (*ACCS2*, *PRKAG1*, *TRMT10A*, and *SLC28A3*) was obtained by RT-PCR, and the desired promoter regions were amplified from chromosomal DNA with the primers listed in Table S1. All the amplified DNA sequences were confirmed by sequencing in Invitrogen company and then cloned into the pGL3 basic vector (Promega, Madison, WI, USA). The CDS of porcine *MAMSTR* was amplified on the basis of the sequence and subcloned into the HindIII-XhoI site of the pCDNA3.1 vector. Plasmids were prepared using Endotoxin-free Plasmid Mini Kit II (Omega, Biel, Switzerland). PK-15 cells were cultured in 96-well plates with DMEM before transfection. The cells were transiently transfected with 0.2 µg of gene-pro-Luc and 0.02 µg of pGL3-TK-luc as normalizing vectors in each well using Lipofectamine™ 2000 reagent (Invitrogen) in Opti-MEM^®^ I medium (Invitrogen). pGL3-Basic and pGL3-Control were used as the negative and positive control, respectively. Growth DMEM was changed at 8 h after transfection. The luciferase activity was determined at 24 h post-transfection with a dual-luciferase assay system (PerkinElmer, Inc., Waltham, MA, USA).

## 3. Results

### 3.1. Effects of MAMSTR Genes on C2C12 Cells

**Figure 1:**
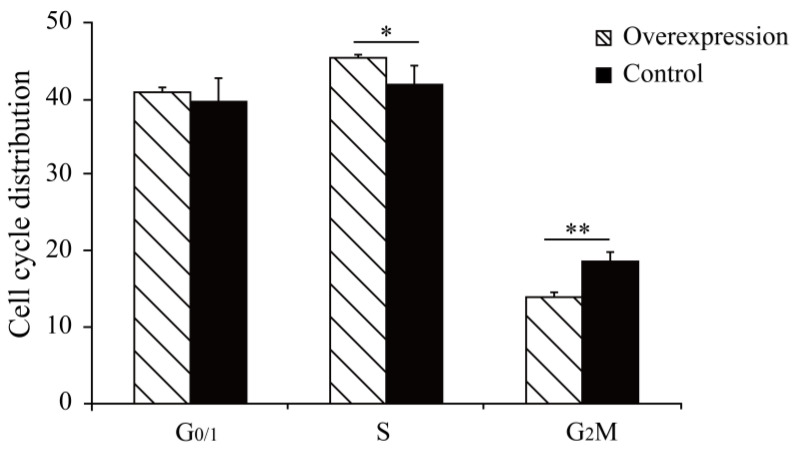
Cell proliferation results of the expression of Mus-*MAMSTR*. * represents significant difference (*p* < 0.05). ** indicates extremely significant difference (*p* < 0.01).

**Figure 2:**
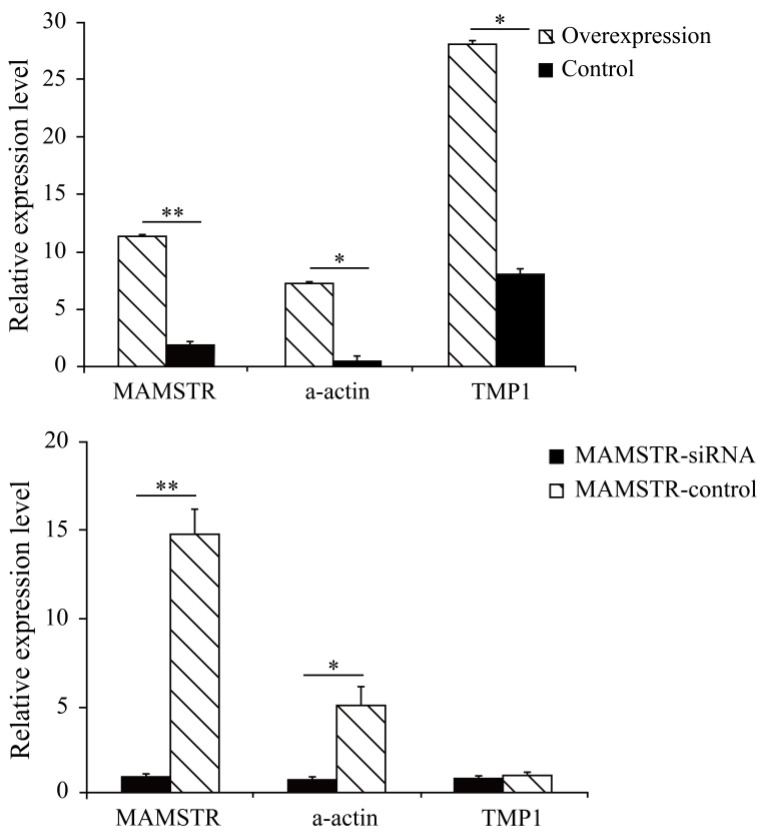
Real-time PCR results after transfection of *MAMSTR* and *MAMSTR*-siRNA. * represents significant difference (*p* < 0.05). ** indicates extremely significant difference (*p* < 0.01).

The results showed that the cell cycle G2/M phase had a proportion of 14.01% after overexpression of the *MAMSTR* gene, the control group was 18.71%, and S stage cells significantly increased after *MAMSTR* gene transfection (*p* < 0.05). Hence, the overexpression of the *MAMSTR* gene promoted the proliferation of C2C12 cells (Figure 1). Then, the expression level of *MAMSTR* before and after overexpression was detected using qPCR. The results showed that the expression level of the *MAMSTR* gene was upregulated and downregulated after the transfection of *MAMSTR* and MAMSTR-siRNA, respectively; the expression levels of muscle growth-related genes A actin and TMP1 were significantly upregulated (*p* < 0.05) after overexpression of the *MAMSTR* gene; the expressions of A actin (*p* < 0.05) and TMP1 (*p* > 0.05) were downregulated after MAMSTR-siRNA transfection (Figure 2). In sum, *MAMSTR* genes could promote the proliferation of C2C12 cells.

### 3.2. Detection of MAMSTR mRNA and Protein Levels after Recombinant Adenovirus Infection

**Figure 3:**
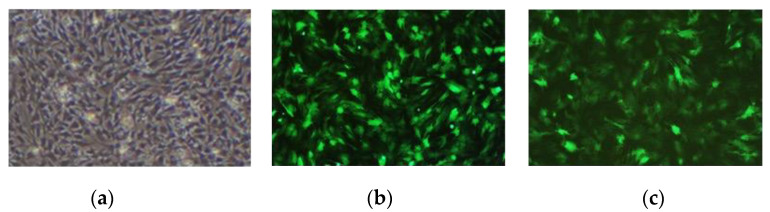
Muscle fibroblasts after recombinant adenovirus infection. (**a**) Control; (**b**) Ad-GFP; (**c**) Ad-*MAMSTR*, 4×.

**Figure 4:**
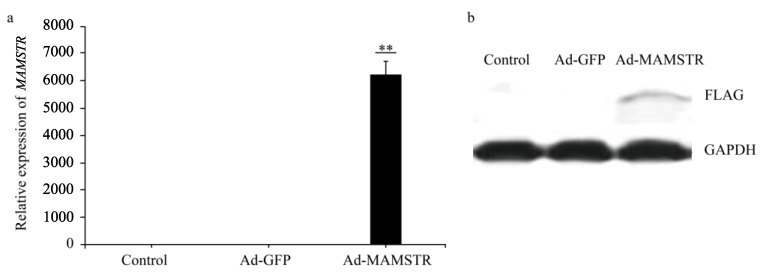
Expression of *MAMSTR* after adenovirus infection. A total of 42 h after transfection, the (**a**) mRNA and (**b**) protein levels of MAMSTR were detected by q-PCR and WB. ** indicates extremely significant difference (*p* < 0.01).

The results observed under a fluorescence microscope are shown in Figure 3. Then, we collected the cells and extracted RNA and protein. The expression of the *MAMSTR* gene was detected using q-PCR and WB with GAPDH as the reference gene. The results indicated that the *MAMSTR* gene was highly expressed in muscle fibroblasts compared with the negative control and empty carrier (Figure 4a,b).

### 3.3. ChIP-Seq Analysis

**Figure 5:**
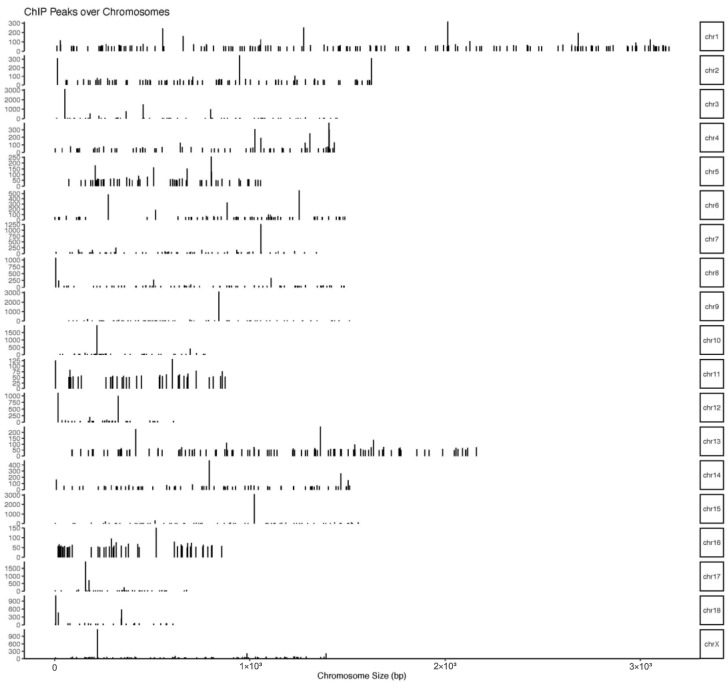
Distribution of *MAMSTR*-binding sites over the chromosomes. The graph represents the distribution of *MAMSTR*-binding sites over the chromosomes. Bars represent significant *MAMSTR* peaks. The length of the bar represents the peak strength.

**Figure 6:**
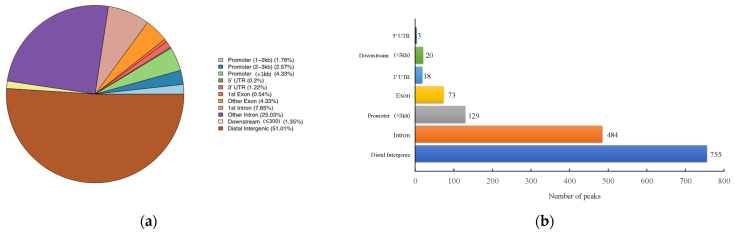
Statistical analysis of peak distribution in different regions. (**a**) Distribution of function regions of different peaks. (**b**) Bar chart of the numbers of *MAMSTR*-binding sites categorized in accordance with the peak location across the pig genome.

**Figure 7:**
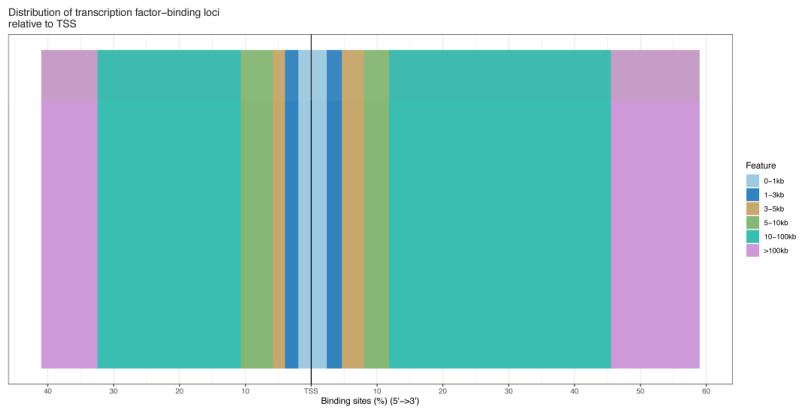
Location distribution of different peaks from TSS.

**Figure 8:**
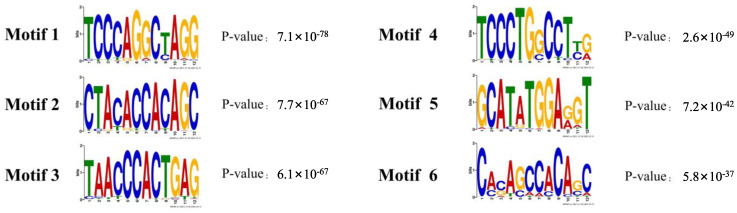
Enriched sequence motifs in *MAMSTR* peaks throughout the genome.

The number of pass filtering reads and uniquely aligned reads is listed in the Table 1. Using a threshold *p*-value < 10^−6^ and an FDR < 20% in the immunoprecipitation versus input comparison, we identified 1506 sites bound by *MAMSTR*. These peaks were annotated to the most proximal TSSs of genes, giving 962 different genes, thus indicating that some genes had more than one proximal *MAMSTR*-binding site. The distribution of *MAMSTR* over chromosomes is shown in Figure 5. *MAMSTR* associated with all chromosomes. In total, 131 sites (10.4%) were located proximal to the TSS region, 382 (30.3%) were in introns, 669 (53.1%) were distal intergenic, 50 (4%) were in exons, 3 (0.3%) were in 5′-UTRs, and 17 (1.4%) were in 3′-UTRs. The remaining nine binding sites (0.7%) were located downstream (Figure 6a,b). The annotation result showed that most of the peaks were 10–100 kb away from TSS (Figure 7). The distribution of these binding sites tended to be at the 3′ end of TSS. To determine whether the DNA sequences associated with *MAMSTR* contained over-represented motifs, these DNA sequences were studied using the MEME program. The analyses revealed five over-represented sequences. The specific structure of motif is shown in the figure below, and the expected value (*p*-value) of motif significance is given (Figure 8).

### 3.4. Function and Pathway Enrichment Analysis of Peak-Associated Genes

**Figure 9:**
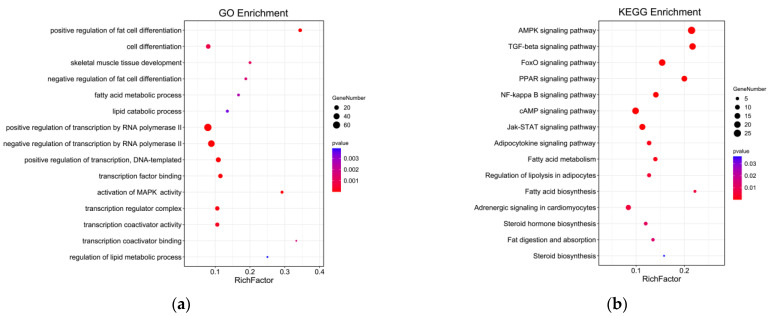
GO and KEGG analyses of genes with *MAMSTR*-binding sites. (**a**) GO enrichment analysis of genes with *MAMSTR*-binding sites. (**b**) Enrichment analysis of genes with *MAMSTR*-binding sites KEGG pathway.

**Figure 10:**
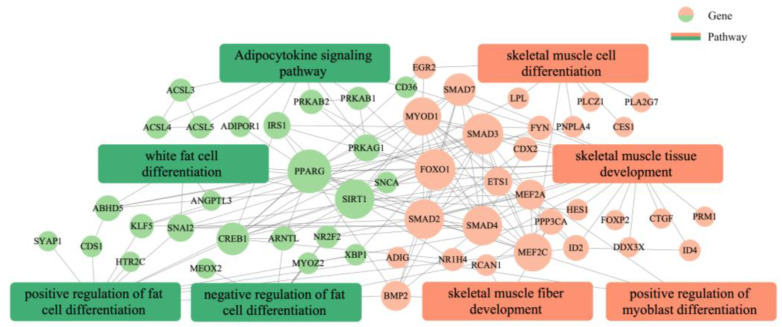
Analysis of the interplay between adipose and skeletal muscle development and differentiation regulation networks.

GO annotation was carried out on the potential target genes. GO and pathway enrichment analyses were also performed, and the results are shown in Figure 9. The most enriched GO terms included transcription regulator complex, activation of MAPK activity, transcription coactivator activity, cell differentiation, positive regulation of fat cell differentiation, skeletal muscle cell differentiation, and white fat cell differentiation (*p* < 0.05). The KEGG enrichment analysis indicated that the target expressed genes were mainly involved in AMPK signaling pathway, TGF-beta signaling pathway, FoxO signaling pathway, fat digestion and absorption, adipocytokine signaling pathway, fatty acid degradation, adrenergic signaling in cardiomyocytes, and fatty acid metabolism (*p* < 0.05). The complete annotated results are shown in Table S2. Most genes involved in the regulation pathway of adipose differentiation and metabolism interacted with those involved in the regulation pathway of skeletal muscle proliferation and differentiation, and some genes were involved in both pathways related to skeletal muscle differentiation and proliferation and pathways related to adipocyte differentiation and metabolism, such as *MEF2A* and *MYOD1*. These results suggested that these genes may play an important role in the regulation of skeletal muscle and adipose differentiation (Figure 10).

### 3.5. Verification and Analysis of Potential Target Genes in the Promoter Region

**Figure 11:**
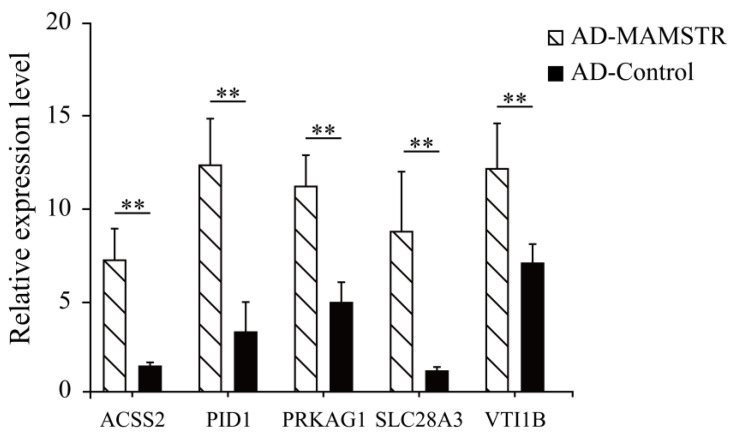
ChIP-qPCR validation results of potential target genes in the promoter region. ** indicates extremely significant difference (*p* < 0.01).

**Figure 12:**
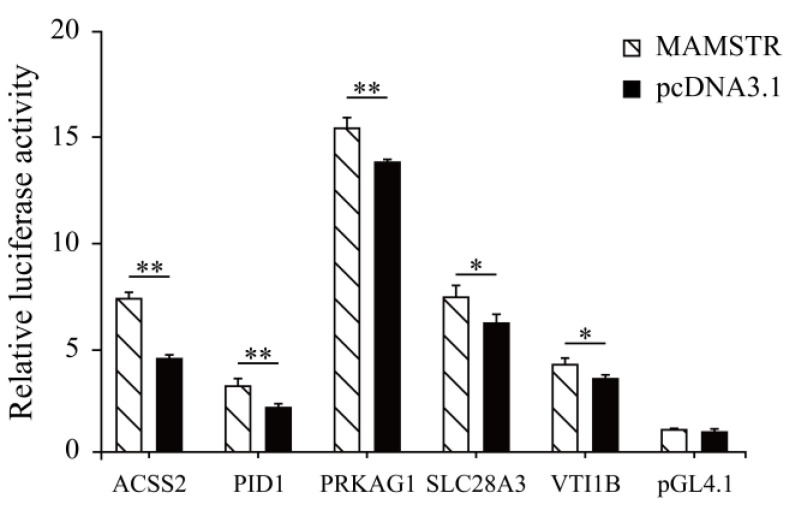
Luciferase reporter assay after co-transfection of the TF *MAMSTR* in PK-15 cell lines. * represents significant difference (*p* < 0.05). ** indicates extremely significant difference (*p* < 0.01).

Thirteen target genes with binding sites located in the promoter region were identified in accordance with the characteristics of TFBSs (Table 2, See Table S3 for the full content). The ChIP-qPCR results indicated that the *PID1*, *VTI1B*, *PRKAG1*, *ACSS2*, and *SLCA328A3* genes were significantly different (Figure 11). To confirm the relationship of *MAMSTR* and its target genes, the promoter activity was assessed after co-transfection via luciferase reporter assay in PK-15 cells. The results showed that it was significantly increased in the transcriptional activity when co-transfected with the promoter of *MAMSTR* gene (Figure 12).

## 4. Discussion

Regulation of gene expression is an important part of functional genomic research. Currently, with the development and improvement of high-throughput sequencing technologies, many projects are gradually being undertaken to study the entire functional genome of muscles [29]. These projects include ENCODE (ENCyclopedia Of DNA Elements) [30] and FANTOM (Functional ANnoTation Of Mouse) [31], among others. Research on the muscle functional genome helps to explore new regulatory mechanisms and biomarkers, thus leading to a deeper understanding of the development and function of complex muscle tissues. Therefore, this study identified *MAMSTR*-binding sites and downstream genes in porcine myofibroblasts by combining experimental and bioinformatic approaches. This is the first report on the role of *MAMSTR*-binding sites in porcine myofibroblasts using the ChIP-Seq technique. In this study, 967 peak-related genes were identified, and the expression levels of *PID1*, *VTI1B*, *PRKAG1*, *ACSS2*, and *SLC28A3* and their transcriptional activities were screened and verified via functional labeling and analysis.

*MAMSTR* is a transcriptional coactivator. It stimulates the transcriptional activity of *MEF2C*, as well as *MYOD1* activity in part via *MEF2*, resulting in the enhancement in skeletal muscle differentiation. The functional analysis of *MAMSTR*-binding sites and downstream genes showed that transcription coactivator activity, transcription coactivator binding, and transcription regulator complex were significantly enriched, which is consistent with the results of previous studies. During skeletal muscle development, the programmed expression of a series of important genes leads to the expression of skeletal muscle-specific genes, which form a complex regulatory network and signal transduction pathway to promote skeletal muscle development in an orderly and controlled manner. TFs are critical in muscle growth and development. Members of the MRF family are typical inducers of skeletal muscle development, including the early MRFs (*MYOD*, *MYF5*, *MYF6*, *MRF4*, etc.) and the late differentiation marker gene (*MyoG*) [11]. The *MEF2* family (*MEF2a*, *MEF2b*, *MEF2c*, and *MEF2d*) is a kind of key TF discovered after *MyoD*, which controls the expression of myogenic genes [13]. The pathway gene interaction network showed that the *MYOD1* and *MEF2A* and *MEF2C* members of the *MRF* family and *MEF2* family were involved in skeletal muscle development and differentiation and interacted with the key genes regulating lipid differentiation. *MYOD1* and *MEF2C* were involved in both positive regulation of myoblast differentiation and negative regulation of lipoblast differentiation. MEF2 proteins were reported to have the potential contributions to adult muscle regeneration. Jin et al. analyzed the promoter of the porcine *MEF2C* gene for further understanding of the *MEF2C* gene. The transcriptional activity of *MEF2C* promoter in differentiated C12C2 cells was found to be higher than that in proliferating C2C12 cells, accompanied by upregulation of *MEF2C* mRNA expression [32]. Previous studies have found that HMG domain protein 20A (*HMG20A*) is highly expressed in the early stage of adipogenic differentiation of porcine intramuscular fat, which may be involved in regulating adipogenesis. Ruixiao et al. confirmed via qRT-PCR and ChP-PCR that *MEF2C* is the true target of *HMG20A*, and that *HMG20A* plays a negative regulatory role through *MEF2C* [33]. Myogenic transdifferentiation can be accomplished through ectopic *MYOD1* expression, which is facilitated by various signaling pathways associated with myogenesis [34]. Myocardial-related TF *MAMSTR* has also been reported to work with *MyoD* to activate skeletal muscle gene expression [15,35]. Zheng et al. found that *MYOD1* inhibited avian adipocyte differentiation via the miRNA-206/*KLF4* axis [36]. The above reports are consistent with the results of our analysis. Thus, *MRF* family members and *MEF2* family genes play pivotal roles in adipose and skeletal muscle tissue development and differentiation.

*PRKAG1* is a protein-coding gene. The protein encoded by this gene is a regulatory subunit of the AMP-activated protein kinase (AMPK). AMPK is a heterotrimer consisting of an alpha catalytic subunit and noncatalytic beta and gamma subunits. AMPK is an important energy-sensing enzyme that monitors cellular energy status. It is a key enzyme involved in regulating de novo biosynthesis of fatty acid and cholesterol. This subunit is one of the gamma regulatory subunits of AMPK. Alternatively spliced transcript variants encoding distinct isoforms have been observed [37]. Demeure et al. showed that *PRKAG1* was mapped near regions containing QTLS for traits that influence obesity [38]. Yan et al. constructed a lentiviral vector carrying shRNA-targeting *AMPKγ.* Treatment with cordycepin (CCS) significantly increased the levels of phosphorylated AMPK in normal cells and decreased the levels of cholesterol and triglyceride. However, in *AMPKγ*-silenced cells, the effect of CCS on AMPK activation and lipid synthesis was almost completely eliminated without changing the expression level of total AMPK or *AMPKγ* protein. These results suggested that the *AMPKγ* gene might be involved in the activation of AMPK by CCS and the regulation of intracellular lipids [39]. *PID1* is a protein-coding gene involved in several processes, including mitochondrion morphogenesis, negative regulation of phosphate metabolic process, and positive regulation of macromolecule metabolic process. The presence of *PID1* in the cytoplasm promotes proadipocyte proliferation and is associated with adipocyte deposition [40,41]. *PID1* can interact with lipoprotein receptor-related protein 1 in adipocytes, but not with insulin receptor. It has also been reported to be a key regulator of glucose metabolism in adipocytes [42]. Yi et al. demonstrated that *PID1* alters insulin antilipolysis and increases lipolysis by inhibiting *AKT/PKA* activation [43]. In addition, some studies have suggested that *PID1* may be involved in adipocyte differentiation [44,45]. In conclusion, *PID1* is widely involved in adipocyte metabolism, proliferation, and differentiation. Acyl-CoA synthetase short chain family member 2 (*ACSS2*) is also a protein-coding gene. This gene encodes a cytosolic enzyme that catalyzes the activation of acetate for use in lipid synthesis and energy generation. The protein acts as a monomer and produces acetyl-CoA from acetate in a reaction that requires ATP [46,47,48]. The interaction of microRNA (miRNA/miR)-15b with the *ACSS2* gene is important for the development of abdominal aortic aneurysm (AAA). Apoptosis of aortic vascular smooth muscle cells (VSMCs) is a pathological feature of AAA. Shujie et al. demonstrated that *ACSS2* may be a direct target of miR-2b-15p, and has-miR-15b-5p can regulate the proliferation and apoptosis of human VSMCs by targeting the *ACSS2*/*PTGS2* axis [49]. In addition, Zhou et al. demonstrated that *ACSS2* can lead to increased muscle atrophy through the metabolic reprogramming of pancreatic cancer [50]. Aside from the abovementioned genes, *VTI1B* and *SLC28A3* can also significantly improve transcriptional activity when co-transfected with *MAMSTR* gene promoters. Nevertheless, due to the lack of relevant studies, *VTI1B* and *SLC28A3* are speculated to be involved in metabolic pathways or the development and differentiation of skeletal muscle cells and adipocytes through other ways.

Results related to *MAMSTR* TFBSs not only provide evidence for its potential role as a key regulator during myofibroblast differentiation, but also shed light on the underlying molecular mechanisms involved. Specifically, our findings suggest that *MAMSTR* TFBSs may act as enhancers or suppressors of certain genes involved in the activation and maintenance of myofibroblasts. By elucidating these regulatory networks, we can gain deeper insights into the complex genetic pathways governing myofibroblast development and provide support for future research.

## 5. Conclusions

In this study, we comprehensively investigated the role of *MAMSTR* in porcine myofibroblasts via ChIP-Seq analysis. Our results related to *MAMSTR* TFBSs represent a better understanding of the regulatory mechanism during myofibroblast development. Specifically, the identification of DNA motifs and regulatory genes provides a new approach to learning the molecular mechanism of *MAMSTR* during myofibroblast development. Overall, these results improve our knowledge of the genetic mechanism underlying the transcriptional regulation and functional characterization of candidate downstream genes involved in the myofibroblast development process.

## Figures and Tables

**Table 1:** Genome alignment distribution of ChIP-Seq data.

Sample Name	Pass Solexa CHASTITY Quality Filter	Aligned (UCSC susScr3)
Ad-GFP-IP	22,346,032	9,131,285
Ad-GFP-Input	26,074,155	19,952,766
Ad-MAMSTR-IP	27,937,935	10,623,701
Ad-MAMSTR-Input	32,682,601	22,873,420

**Table 2:** Analysis of TFBSs in the promoter region (TOP 10).

Refseq_Name	Symbol	Chr	TSS	TTS	Strand	Chr:Start–End	Length
NM_001143695	*ACSS2*	chr17	43455187	43510660	+	chr17:43456618-43456877	260
NM_214349	*PC*	chr2	4605274	4609731	+	chr2:4603905-4604407	503
NM_001098594	*MLPH*	chr15	151614165	151614293	+	chr15:151613849-151614361	513
NM_001001642	*PRKAG1*	chr5	15522471	15443954	−	chr5:15520284-15520881	598
NM_001173520	*PID1*	chr15	144255955	144256107	+	chr15:144254596-144255197	602
NM_001244146	*TRMT10A*	chr8	130101783	130133413	+	chr8:130100338-130100970	633
NM_001098585	*AP3B1*	chr2	88719584	88534907	−	chr2:88719756-88720316	561
NM_001113702	*SLA-2*	chr7	24687093	24642158	−	chr7:24688625-24689126	502
NM_214437	*VTI1B*	chr7	97720791	97693163	−	chr7:97719717-97720147	431
NM_001195370	*SPIN1*	chr1	32595576	32594555	−	chr1:32594480-32595251	772

## Data Availability

All data generated or used during this study are available upon request from the corresponding author.

## Supplementary Materials

The following supporting information can be downloaded at: https://www.mdpi.com/article/10.3390/ani13111731/s1, Table S1: The promoter primer sequence of potential target gene; Table S2: GO and KEGG analyses of genes with *MAMSTR*-binding sites; Table S3: Analysis of TFBSs in the promoter region.
